# Draft Genome Sequence of Lactobacillus crispatus Strain V4, Isolated from a Vaginal Swab from a Young Healthy Nonmenopausal Woman

**DOI:** 10.1128/MRA.00856-19

**Published:** 2019-09-19

**Authors:** Maximilien Clabaut, Amine M. Boukerb, Pierre-Jean Racine, Chantal Pichon, Coralie Kremser, Aïna Queiroz, Madina Karsybayeva, Gérard Redziniak, Sylvie Chevalier, Marc G. J. Feuilloley

**Affiliations:** a Laboratoire de Microbiologie Signaux et Microenvironnement (LMSM) EA 4312, Université de Rouen Normandie, Normandie Université, Évreux, France; b Centre de Biophysique Moléculaire, CNRS UPR4301, Orléans, France; c GimoPharm, Longjumeau, France; d Seqens, Limoges, France; e Remedials Laboratoire, Paris, France; f Cosmetic Inventions, Antony, France; Loyola University Chicago

## Abstract

Lactobacillus crispatus strain V4 was isolated from a vaginal swab from a healthy nonmenopausal 35-year-old French woman. We report here its draft genome sequence of 2,091,889 bp, with an average G+C content of 37.02%.

## ANNOUNCEMENT

Lactobacillus crispatus is one of the dominant lactic acid bacteria (LAB) colonizing the healthy vagina. This bacterium is essential in the maintenance of a healthy vaginal epithelium (1). L. crispatus is known to produce antimicrobial compounds, including organic acids, hydrogen peroxide, bacteriocins, and biosurfactants (2). It is also able to modulate the secretion of cytokines and chemokines that enhance the immune response of vaginal epithelial cells (3). Adhesion and establishment of L. crispatus on the human vaginal epithelium are prerequisites for its protective role (4). In the first step, L. crispatus autoaggregates, leading to the formation of microcolonies on the vaginal epithelium, which then evolve into biofilms, covering the whole ecological niche and providing protection against pathogens (5).

L. crispatus V4 was isolated from a vaginal swab from a young healthy nonmenopausal woman in 2018. These bacteria were collected under the control of the CRO Bio-EC (Longjumeau, France) in agreement with French and European Union ethics guidelines (ARS Biomedical Research agreement 2012-12-010 and Bioethic agreement DC-2008-542). Bacterial isolates were grown anaerobically onto de Man, Rogosa, and Sharpe (MRS) agar plates overnight at 37°C (ISO, VWR reference 84607.0500). Purified bacterial strains were obtained by single-colony isolation. V4 isolates were cultured and later maintained at −80°C using MRS with 20% glycerol.

In the first step, L. crispatus strain V4 was identified with total proteome analysis using a Bruker Autoflex III matrix-assisted laser desorption ionization–tandem time of flight (MALDI-TOF/TOF) mass spectrometry instrument coupled to the Biotyper software (6). Subsequently, 16S rRNA sequencing was performed, and the BLAST algorithm allowed sequence comparison with other L. crispatus strains reported in the NCBI 16S RefSeq database. Since the 16S rRNA sequence of strain V4 displayed 100% sequence identity with those of L. crispatus strains CO3MRSI1 and AB70 (GenBank accession numbers CP033426 and CP026503, respectively), strain V4 was identified as belonging to the L. crispatus species.

Genomic DNA was extracted from MRS broth L. crispatus V4 culture by treating cells with a lysozyme solution (20 mM Tris-HCl [pH 8.0], 2 mM EDTA, 1.2% Triton X-100, 20 mg/ml lysozyme), followed by treatment with a GeneJET genomic DNA purification kit (catalog number K0721; Thermo Fisher Scientific). Genomic libraries were prepared with the Nextera XT DNA sample preparation kit (Illumina) and sequenced using v3 chemistry and 2 × 250-bp paired ends on an Illumina MiSeq platform following the manufacturer’s instructions (LMSM Évreux, Rouen Normandy University).

Trimmomatic v.0.36 (7) was used to trim the 2,316,108 generated reads. FastQC v.0.11.6 (8) was used to check their quality. Genome assembly was achieved *de novo* with Unicycler v.0.4.7 (9) with default parameters. Quast v.5.0.0 (10) was used to check the consistency of the obtained assembly (i.e., genome size, number of contigs, *N*_50_, and G+C content). The final assembly was annotated using Prokka v.1.13.4 (11).

The assembled genome consisted of 252 contigs (coverage, 553×) with a genome size of 2,091,889 bp and an average G+C content of 37.02%, which was consistent with already published L. crispatus genome sequences. The largest contig size was 102,546 bp, with an *N*_50_ value of 18,575 bp. The draft genome harbors 2,177 protein-coding sequences, 65 tRNAs, and 3 rRNA gene clusters. The annotated sequences allowed the identification of putative virulence and antibiotic resistance genes relevant to *Lactobacillus* spp. (12). Subsequent analyses involving comparative genomics will be undertaken in an upcoming study.

Altogether, the availability of the genomic information from strain V4 will improve our understanding of the genetic diversity within the species and the mechanisms involved in microbiome-vaginal health interactions.

### Data availability.

The draft genome sequence of Lactobacillus crispatus V4 has been deposited at DDBJ/ENA/GenBank under the accession number SRLG00000000. The version described in this paper is the first version, SRLG01000000. The raw sequencing data have been deposited in the same database under the accession number SRR8842498.
